# Alcohol and Physical Deterioration

**Published:** 1908-02-15

**Authors:** 


### ALCOHOL AND PHYSICAL DETERIORATION.

Mr. W. McAdam Eccles, M.S., F.R.C.S., delivered, in the Town Hall, Oxford, on February 4, the Lees and Raper memorial lecture, the subject being "The Relation of Alcohol to Physical Deterioration and National Efficiency." Professor Osler presided. The lecturer said that soon after the appearance of life upon earth it was subjected to a struggle for existence, chiefly by reason of irritation by poisons around it. One of those was alcohol, a liquid which was very abundant in nature. Its action upon living tissue tended to produce deterioration. If this was its action upon the cell, it would be its action on the individual; and if upon the individual, then also upon the race. A child might be thus affected even before birth. Many experiments had been made which proved that alcohol was most deleterious in the early development of the animal, whether it were a tadpole, a chick, a puppy, or a child. The effect of alcohol given to infants, either through their mothers' milk or by the spoon, was so bad that many hospitals issued specific warnings concerning it. Alcohol was a selective poison—that was to say, it picked out one tissue for deterioration more than another, and one individual to a greater degree than his neighbour. Alcohol acted harmfully upon the cells of the blood and the cells of the blood vessels, so that the prolonged repetition of even moderate doses resulted in serious inefficiency of their functions. This was forcibly evidenced by certain life-insurance statistics. Physical deterioration led necessarily to inefficiency, and inefficiency of the nation must inevitably lead to loss of supremacy. Some measures were therefore not only desirable but imperative to stem the tide which appeared to be flowing over the land. To attain this end various suggestions were put forward, but the lecturer urged that the instruction of the child in the elementary school was of the utmost importance. The elements of hygiene and temperance were vital, and they could be made attractive, even to the very young, if inculcated by a teacher of experience and ability. When a child had been taught those principles and had reached an age of discretion, it should not be unduly tempted to fall from right paths by the continuance of a public-house at every street corner. But if the public-house went, something should take its place. Still further to alter the trend of public opinion the Universities should themselves encourage research into the many problems involved in the question of alcohol, and publish the results with their weight of authority. By the establishment of a sensible and firm public opinion on the matter nearly all the difficulties of politicians and social reformers would disappear.

